# Personal and professional quality of life among French health care workers during the first COVID-19 wave: a cross-sectional study

**DOI:** 10.1186/s12912-022-00860-y

**Published:** 2022-04-07

**Authors:** Armand Grelier, Olivia Guerin, Fathia Levavasseur, Frédérique Caillot, Jacques Benichou, François Caron

**Affiliations:** 1 CHU Rouen, Department of Infectious Diseases, F-76000 Rouen, France; 2 CHU Rouen, Department of Biostatistics and Clinical Research, F-76000 Rouen, France; 3 CESP U 1018 Inserm High-Dimensional Biostatistics for Drug Safety and Genomics, Université Paris-Saclay and Université de Rouen, Rouen, Normandie France; 4 DYNAMICURE Inserm UMR 1311, Université de Rouen and Université de Caen, Rouen, Normandie France

**Keywords:** Quality of life, Encouragements, Health care workers, COVID-19 pandemic

## Abstract

**Background:**

We aimed to assess the personal and professional quality of life changes among health care workers of different professions during the COVID-19 pandemic in a large French university hospital. Other published data originated from countries with different health care systems and outbreak dynamics.

**Methods:**

All health care workers from our hospital were invited to fill-in an anonymous e-questionnaire of 71 questions regarding perceived personal, professional and overall quality of life before and during the first COVID-19 wave, general profile, occupation and job characteristics, change of assignment, COVID-care features if relevant, general perception during the first wave, and personal experience of being encouraged or stigmatised.

**Results:**

There were 794 participants, with a majority of nursing professionals (*n* = 416, 56%), including 57 nurse managers, 243 nurses, and 116 nurse assistants. Other participants were physicians (*n* = 188) and other health care staff (*n* = 140). Before the crisis, professional quality of life was low (6.5 on a 10-point scale) overall. The personal quality of life was higher (8.1) particularly for physicians and nurse managers. The COVID crisis saw a marked decrease in the personal quality of life (− 1.7), more pronounced in younger health care workers. Professional quality of life was less affected (− 0.4) and stayed almost constant for physicians. Staff in COVID units had a more positive perception of the crisis but experienced more fatigue, which resulted in similar quality of life levels in COVID and non-COVID units. Encouragements originated more often from relatives or colleagues than hospital managers and were exceptionally common: 63.4% of all participants, from 50.5% for other staff to 71.3% for physicians (*p* = 0.0005). Stigmatisation was reported by 19.3% of participants, with a higher proportion (*p* = 0.0001) among nurses (26.3%) and assistant nurses (23.3%) than among physicians (8.5%). From multivariate analysis, higher age, working as a physician and receiving encouragements were independently associated with lower loss of overall quality of life.

**Conclusions:**

The resilience of health care workers was high overall during the first COVID wave although the quality of life decreased more among nursing staff. Social support in the form of encouragements is a key part of management, particularly in times of crisis.

## Background

Since the first trimester of 2020, the COVID-19 pandemic has disrupted the personal and professional lives of everyone worldwide, especially for health care workers (HCWs). This was particularly the case during the period referred to as the ‘first COVID-19 wave’ which led to a drastic lockdown in many countries. In France, from March to May 2020 schools and most shops were closed, travel limited and hospitals deserted because of fear of contamination when masks were not available for the general population.

On the front line of the fight against the pandemic, hospital HCWs experienced very diverse situations. Some remained assigned to their unit whereas others moved from their regular unit to another mainly to take part in COVID care. Personal situations were equally diverse: lockdown could be perceived as difficult or more tolerable depending notably on one’s age, material and personal conditions, the encouragements one received (e.g., ‘*the 8 o’clock applause*’) or, conversely, the stigmatisation from which one suffered (e.g., ‘*the nurse next door is going to infect us’*). All these factors have certainly resulted in experiences of the COVID-19 pandemic markedly different from one HCW to another but there have been very few reports so far on the quality of life of HCWs during the COVID-19 pandemic.

Our aim was to assess level of change in personal and professional quality of life during the first wave of the COVID-19 pandemic as compared to the pre-COVID period and determinants of quality of life change through a survey conducted among HCWs from Rouen University Hospital, a large hospital located in the Normandy region of Northwestern France.

## Methods

The Quali-CoV-H was an observational, cross-sectional, monocentric study, that used an anonymous self-assessment questionnaire offered to all staff at Rouen University Hospital. HCWs were defined as staff delivering care either directly (physicians, nurses…) or indirectly (supervisors, laboratory technicians …).

The questionnaire (in French) was published and the data were collected through the ‘Lime Survey’ application (http://www.limesurvey.org/). Workers could learn about the study through at least one of the following channels. The questionnaire was uploaded online on the first page of Rouen University Hospital Intranet website, was emailed to all e-mail addresses of the hospital, and was shared on the hospital Facebook page, alongside a link to download the application for those who wished to complete the survey on their smartphones. The survey typically took 10 to 15 min to complete (depending on time spent on responses in open full text). There were 71 questions that assessed each participant’s general profile including personality traits, housing and commute to work; occupation and job characteristics including working in night shifts; quality of life ‘before COVID-19’; change of assignment; quality of life during the ‘first wave’; COVID-care features if relevant; general perception including professional empowerment, level of implication in the fight against COVID-19, knowledge about the epidemic, fatigue, stress; and personal experience of being either encouraged or stigmatised. Most questions were of a categorical nature, a few were quantitative and 17 used an integer scale from 0 to 10, 0 being the worst level. Five optional questions allowed open responses in full text. Both personal and professional dimensions of quality of life were assessed. For both dimensions, material and relational quality of life were each graded from 0 to 10 on an integer scale, 0 and 10 standing for the worst and best possible levels, respectively, and then were averaged to obtain an overall score for each dimension. Finally, overall quality of life was obtained as the average of personal and professional scores. Descriptive analyses were performed overall and by occupation, hospital unit type (COVID ward, non COVID ward), and change of assignment during the first wave. Only occupations which accounted for at least 7% of the participants were analysed separately, while the others were the subject of grouping.

Quality of life scores and absolute change in quality of life scores from baseline to the COVID-19 phase were compared between categories of variables using the Student t-test or one-way ANOVA as appropriate. Comparisons of quality of life between the periods before and during the first wave were performed using Student’s paired t-test. Dichotomous variables (e.g., having been encouraged or stigmatised) were compared among categories of other variables using Pearson’s chi-square test or McNemar’s test as appropriate. Multiple linear regression was used to assess factors independently associated with change in the quality of life. The adjusted multiple linear regression model included sex, age category, occupation, type of unit, type of assignment, night work, receipt of encouragements and experience of stigmatisation, allowing for mutual adjustment. We checked that validity assumptions were met for all fitted multiple linear regression models, namely independence of residuals, normally distribution and constant variance (homoscedasticity). Furthermore, there were no significant outliers in any of the models.

All statistical tests used two-sided 0.05 significance threshold. All statistical analyses were performed using SAS software, version 9.4, (SAS Institute, Cary, NC).

The free-form items were the subject of a synthesis that presented the most recurring and salient phrases.

The study was performed in accordance with the relevant guidelines and regulations. According to French law relative to clinical research (“Loi Jardé” effective since November 2016), the present study was in the field of human and social sciences, and did not require the approval of an ethics committee. Participation to the study was on a voluntary basis after receipt of information on the study. The questionnaire was anonymous and did not include any personal information. At any time during the online survey completion, participants could decide to stop completion without validating their questionnaire and this ensured that they would not be included in the study and their data would not be recorded. Conversely the final click validated the inclusion in the study.

## Results

The survey took place between May 7th and June 5th, 2020, i.e., at the time the national lockdown (March 17th to May 11th) was ending and the first wave was strongly receding. Indeed, in our hospital the first wave peaked on March 31th (138 hospitalized patients among whom 47 in intensive care units [ICUs]), had decreased to a third by May 7th (46 hospitalized patients, including 25 in ICUs) and to a tenth by June 5th (11 hospitalized patients, including 1 in ICU). A total of 794 participants responded and completed the whole questionnaire, the vast majority of them being HCWs, representing approximately 11% of the HCW staff in Rouen University Hospital. Participants had a mean age of 41 years (range 20–67 years) and were predominantly female (80.0%). The sample included 188 physicians (including 12 residents), accounting for 23.7% of the study sample, 57 nurse managers (7.2%), 243 nurses (30.6%), 116 nurse assistants (14.6%) and 190 other staff (23.9%). Participants were evenly distributed between COVID-related activities (47.9%) and non-COVID-related activities (52.1%). HCWs who changed assignment to reinforce other teams accounted for more than a quarter (27.7%) of the overall sample. Among the 220 participants who changed assignment, more than two-thirds (156, 70.9%) were assigned to COVID units. Compared to staff in the usual assignment, they displayed a significant (*p* < 0.0001) overrepresentation of nurses (43.6 versus 25.6%) and nurse assistants (22.3 versus 11.7%) and, conversely, an underrepresentation of physicians (12.3 versus 28.0%) and nurse managers (2.3 versus 9.1%).

**Fig. 1:**
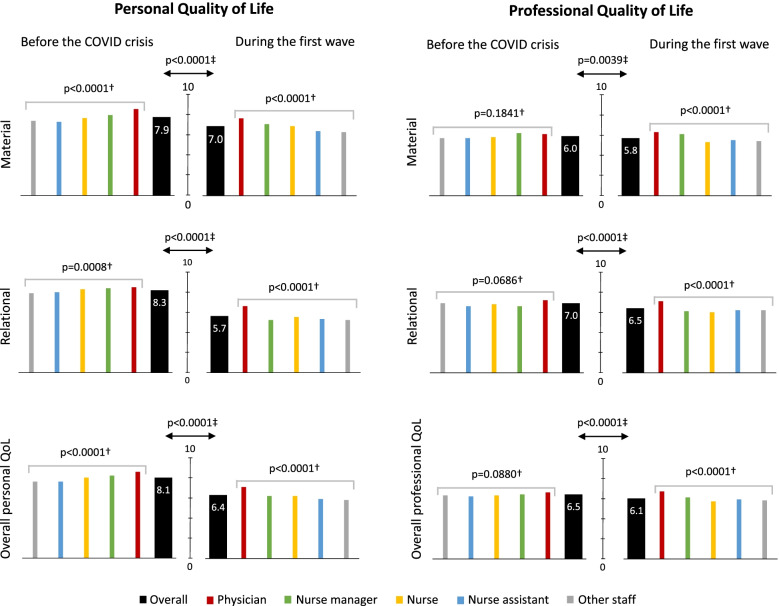
Quality of life before the COVID-19 crisis and during the first wave according to occupation. †From one-way ANOVA. ‡ From Student’s paired t-test. QoL: Quality of life assessed on 0–10 integer scale (for material and relational QoL) or averaged between material and relational QoL (for overall QoL)

The average quality of life scores are shown in Fig. 1 for the total sample as well as according to occupation. For the period preceding the COVID-19 crisis, marks were significantly higher from the personal than professional standpoint, the overall average marks being 8.1 (on a 0–10 integer scale) and 6.5 respectively, hence a 1.6 discrepancy. For most of the items, there was a hierarchy in the results according to occupation: physicians expressed higher marks than nurse managers, who themselves expressed more positive opinions than the nursing staff. Only the assessment of material professional life did not abide by this hierarchy, showing very similar results according to occupation.

The crisis diminished existing differences as it showed an overall sharp decline in personal quality of life, whereas professional quality of life dropped much less so that the discrepancy between personal and professional quality of life dimensions was reduced to 0.3 (6.4 vs. 6.1 for the overall mark) during the first wave period. Alteration of personal quality of life was much more pronounced for the relational dimension (2.6 mean decrease) than for the material one (0.9 mean decrease), and occurred regardless of occupation. Material professional quality of life was almost not altered during the crisis, with a 0.2 mean decrease only (5.8 against 6.0 prior to the crisis, *p* = 0.0039). Various free-form comments illustrated some of the difficulties encountered by participants: *‘lack of equipment’*, *‘increase of the workload’*, *‘adaptation in the face of an unprecedented situation’*, *‘heightened mental and physical burden’*, *‘changing protocols’*, *‘disturbed organisation’*, *‘feeling of unequal distribution of equipment’*; however, other comments highlighted positive aspects: *‘larger number of medical staff*, *‘more beds available due to the decline in activity of certain units’*, *‘improvement of work conditions when shared bedrooms became single ones’*, *‘better traffic and parking’*, *‘comfortable work conditions when needed equipment is fully available’*.

Professional quality of life underwent a more statistically visible fall in its relational aspects, albeit with a limited 0.5 mean decrease (6.5 against 7.0 prior to the crisis, *p* < 0.0001), which differed significantly between occupations (*p* = 0.0088), with almost no change for physicians (− 0.1) in contrast with all other participants. Numerous free-form comments were expressed, many of them more critical of the general context rather than the hospital organisation: *‘feeling of isolation (lockdown, restructuration)’*, *‘poor morale’*, *‘loss of shared activities’*, *‘reorganisations perceived as provoking anxiety’*, *‘general situation as a source of stress’*, *‘oppressive atmosphere’*, *‘exacerbation of conflicts’*, *‘heavy mental load, pressure, tension, concern, prevailing anxiety’*, *‘communication disrupted by social distancing, masks, the limitation of meetings’*, *‘shaken private life’*, *‘disruption of habits and professional routine’*, *‘feeling of regression’*, *‘last-minute reorganisation’*, *‘feeling of lack of good listening from management’.* Nevertheless, some comments were more positive and stressed an increased sense of cohesion and mutual support: *‘inter-unit support’*, *‘deepening of relationships’*, *‘solidarity, collective and institutional spirit, mutual assistance, cohesion within the team’*, *‘the staff was able to adapt and react effectively’*, *‘the crisis period enhanced the relationships between patients and staff’*.

Table 1 reports various features of personal perception during the crisis. Marked differences were observed for most items according to assignment to a COVID unit. Overall, staff in COVID units had a markedly more positive perception of the crisis, notably with regard to their involvement, sense of self-worth, knowledge, effective protection or general comfort in managing the crisis. This was at the expense of experiencing slightly more fatigue but no sense of increased hardship was found.

**Table 1:** Perception of the COVID-19 crisis for all participants and by type of hospital unit

	Overall (*n* = 794)	By type of unit		
		COVID unit (*n* = 380)	Non COVID unit (*n* = 414)	p^a^
Involvement in managing the crisis^b^ (mean)	6.8	7.6	6.2	**< 0.0001**
Sense of self-worth^b^ (mean)	5.4	5.9	4.9	**< 0.0001**
Increased fatigue^b^ (mean)	6.2	6.4	6.0	**0.0149**
General hardship^b^ (mean)				
- personal standpoint	5.0	4.9	5.1	0.0934
- professional standpoint	4.7	4.8	4.6	0.4226
Satisfactory knowledge of COVID-19 risk management^b^ (mean)	6.8	7.1	6.6	**0.0009**
Being comfortable with COVID-19 risk management^b^ (mean)	6.4	6.8	6.1	**< 0.0001**
Feeling protected in the management of COVID-19 risk^b^ (mean)	6.5	6.8	6.2	**< 0.0001**
Receipt of direct encouragements (percentage)	63.4%	71.8%	55.6%	**< 0.0001**
Experience of stigmatisation (percentage)	19.3%	24.0%	15.0%	**< 0.0001**

^a^From Pearson’s chi-square test or Student’s t-test as appropriate

^b^Integer scale from 0 to 10, 0 being the worst score except for ‘increased fatigue’ and ‘general hardship’

**Fig. 2:**
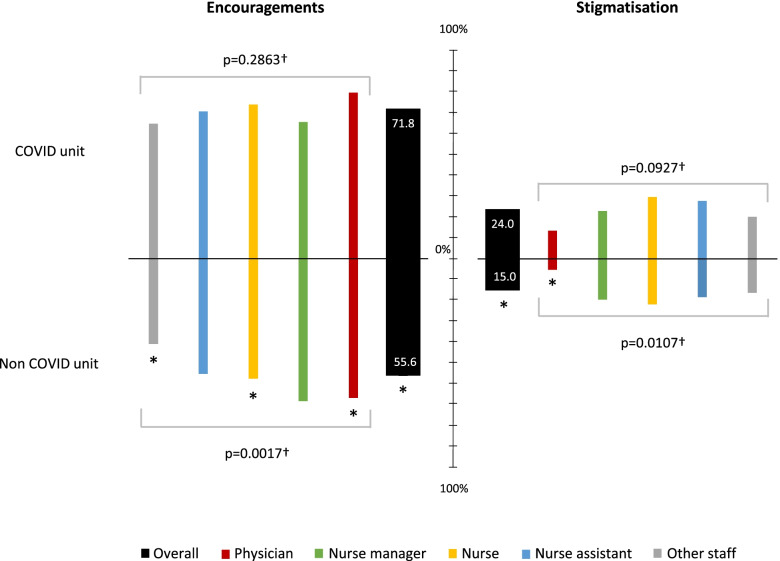
Encouragements and stigmatisation according to occupation during the COVID-19 first wave. †From one-way ANOVA. * *p* < 0.05 for comparisons between COVID and non-COVID units from Student’s t-test

As illustrated in Fig. 2 and Table 1, staff in COVID units was simultaneously more encouraged and stigmatised. Overall, 63.4% of participants reported receiving encouragements, going from 71.3% for physicians to 50.5% for other staff (Table 2), a difference that was significant overall (*p* = 0,0005) among occupations. Stigmatisation was reported by 19.3% of participants with differences according to occupation (*p* = 0.0001), a higher proportion being observed among nurses (26.3%) and assistant nurses (23.3%) whereas few physicians reported experiencing stigmatisation (8.5%). Encouragements took multiple forms - notably through material aid - and originated more often (*p* < 0.0001) from colleague, family members or neighbor than from hospital manager.

**Table 2:** Receipt of encouragements and experience of stigmatisation for all participants during the COVID-19 first wave

	Encouragements (%)				Stigmatisation (%)			
	Overall	COVID Unit	Non COVID Unit	p^a^	Overall	COVID Unit	Non COVID Unit	p^a^
	(*n* = 794)	(*n* = 380)	(*n* = 414)		(*n* = 794)	(*n* = 380)	(*n* = 414)	
**Type of assignment**								
Usual	61.9	72.8	54.9	**< 0.0001**	16.9	21.9	13.7	**0.0109**
Reinforcement	67.3	70.5	59.4	0.1098	25.5	26.9	21.9	0.4350
**Occupation**								
Physician	71.3	79.5	66.1	**0.0484**	8.5	13.7	5.2	**0.0422**
Nurse manager	66.7	65.4	67.7	0.8508	21.1	23.1	19.4	0.7314
Nurse	66.7	73.8	56.9	**0.0058**	26.3	29.8	21.6	0.1512
Nurse assistant	62.9	70.5	54.6	0.0758	23.3	27.9	18.2	0.2177
Other staff	50.5	64.6	40.5	**0.0011**	17.9	20.3	16.2	0.4743
**Emitter**^b^								
Hospital manager	22.0	28.4	16.2	**< 0.0001**	N/A			
Colleague	29.0	39.7	19.1	**< 0.0001**	N/A			
Family member	60.5	69.2	52.4	**< 0.0001**	7.9	9.7	6.3	0.0718
Neighbor	38.8	41.3	36.5	0.1619	9.3	12.1	6.8	**0.0097**
Other	47.1	52.6	42.0	**0.0028**	14.7	18.2	11.6	**0.0091**

^a^From Pearson’s chi-square test

^b^Several types of emitters were possible

*N/A* Not applicable

Table 3 reports on potential determinants of quality of life change between the pre-crisis period and the COVID-19 first wave. From multivariate analysis, three variables appeared independently associated (albeit moderately so) with change in the quality of life: higher age correlated with a lower decline in personal and overall quality of life. Being a physician was associated with little or no change in the quality of life, whether professional or general compared to nurse managers or members of the nursing staff who experienced more decrease in the quality of life. Receiving encouragements was associated with lesser alteration of professional or general quality of life.

**Table 3:** Determinants of change in quality of life during the COVID-19 first wave

	Differences (∆) in QoL^a^ between the periods before and during the first wave								
	Personal QoL			Professional QoL			Overall QoL		
	∆	crude p^b^	adjusted p^c^	∆	crude p^b^	adjusted p^c^	∆	crude p^b^	adjusted p^c^
**Sex**									
Male (*n* = 159)	−1.5	0.0916	0.2352	−0.2	0.2270	0.9628	−0.8	**0.0491**	0.3686
Female (*n* = 635)	−1.8			−0.4			−1.1		
**Age category (years)**									
< 30 (*n* = 113)	−2.2	**0.0026**	**0.0037**	−0.4	0.2015	0.3591	−1.3	**0.0038**	**0.0163**
[30–35) (*n* = 142)	−1.7			−0.4			−1.1		
[35–40) (*n* = 118)	−1.7			−0.2			− 0.9		
[40–45) (*n* = 110)	−2.1			−0.4			−1.3		
[45–50) (*n* = 110)	−1.6			−0.1			−0.9		
[50–55) (*n* = 91)	−1.5			−0.6			−1.1		
≥ 55 (*n* = 109)	−1.1			−0.2			− 0.7		
**Occupation**									
Physician (*n* = 188)	−1.4	0.1927	0.5270	+ 0.1	**0.0002**	**0.0011**	−0.7	**0.0010**	**0.0390**
Nurse manager (*n* = 57)	−2.0			−0.3			−1.1		
Nurse (*n* = 243)	−1.8			−0.6			−1.2		
Nurse assistant (*n* = 116)	−1.8			−0.3			−1.0		
Other staff (*n* = 190)	−1.8			−0.5			−1.2		
**Type of unit**									
COVID (*n* = 380)	−1.8	0.1100	0.1505	−0.3	0.2265	0.0835	−1.1	0.5840	0.8872
Non COVID (*n* = 414)	−1.6			−0.4			−1.0		
**Type of assignment**									
Usual (*n* = 574)	−1.7	0.8520	0.2709	−0.3	0.3006	0.4542	−1.0	0.6394	0.6634
Reinforcement (*n* = 220)	−1.7			−0.4			−1.1		
**Night work**									
Yes (*n* = 314)	−1.7	0.5938	0.9054	−0.4	0.8687	0.1314	−1.0	0.7551	0.4466
No (*n* = 480)	−1.7			−0.3			−1.0		
**Receipt of encouragements**									
Yes(*n* = 503)	−1.7	0.3696	0.1781	−0.2	**0.0082**	**0.0422**	−0.9	**0.0302**	**0.0280**
No (*n* = 291)	−1.8			−0.5			−1.2		
**Experience of stigmatisation**									
Yes (*n* = 153)	−1.9	0.3470	0.6154	−0.4	0.8655	0.8083	−1.1	0.3961	0.7991
No (*n* = 641)	−1.7			−0.3			−1.0		

^a^QoL: Quality of life on 0–10 integer scale

^b^From Student’s t-test or one-way ANOVA as appropriate

^c^From multiple linear regression including all variables in the table and Wald’s test

## Discussion

This first finding of this study is a more negative perception of professional than personal quality of life among HCWs prior to the COVID-19 crisis. At 6.5 out of 10, the mean professional quality of life score was consistent with mean values reported by two previous French surveys which similarly used a self-evaluation on a 0–10 integer scale, namely 5.3 for a study carried out during years 2015–2017 that included 9100 participants across 40 health facilities [1], and 6.1 out of 10 in a 2019 survey of 161 nurses from one hospital [2]. Additionally, a French interprofessional study that used an index number from 0 to 1 reported in 2018 that the professional quality of life of HCWs was lower (0.73) than in many other professional sectors such as the food-processing (0.77) or the energy and environment industry (0.85) [3].

In the period before the crisis, while personal quality of life of participants was positively correlated with their socioeconomic status, professional dissatisfaction was shared widely among all professions and concerned material and relational aspects equally. The current survey was carried out in a context of global and multifactorial *malaise* of the French health care system that has affected caregivers and physicians for years, particularly in public hospitals. This *malaise* reached a peak at the beginning of 2020 [4] right before the COVID-19 crisis began. Subsequently, a modernisation plan including salary increases was announced during the first COVID-19 wave in order to make hospital jobs more attractive [5].

Another major result of this study is the overall high resilience of hospital HCWs during the first COVID-19 wave, with only a slight decline in reported professional quality of life (− 0.4 point on average) compared to the marked drop in their personal quality of life (− 1.7 points on average). However, while physicians reported not being very affected by the COVID-19 crisis in their professional quality of life, the nursing staff expressed more suffering. This socio-professional hierarchy was clearly outlined in the multivariate analysis, with a more marked drop in overall quality of life among the nursing staff independently of age or having received encouragements. Being assigned to a COVID unit even as temporary reinforcement did not seem to have an impact on job satisfaction. Improvement in risk management and satisfaction over becoming involved in the general effort seemed to compensate for the added fatigue.

Our results are consistent with those from studies that assessed the quality of life among HCWs during previous emerging virus outbreaks, showing that younger age and nurse occupation increase the risk of adverse psychological outcomes while a managerial role and receiving social support from peers or family decrease this risk [6]. Our results could also be compared to those from studies performed during the first COVID-19 wave in other countries. In the regions of the world where the epidemic was very strong such as the province of Wuhan in China [7], the Veneto region in Italy [8] or the Basque country in Spain [9] an excess incidence of professional burnout was described, in particular among front-line nurses such as those in ICUs. Among dieticians in Brazil, having a high family income and a teaching practice was correlated with a better quality of life perception, both before and during the wave [10]. In other countries such as Malaysia, where the first wave remained limited, the quality of life of 389 university-based HCWs from different occupations (detail not reported) during the first wave has been reported similar to that prior to the pandemic, with greater perceived social support being strongly associated with a better score [11]. In Australia where the number of COVID-19 cases during the first wave was low with no strong impact on the healthcare system, quality of life was better among HCWs than for the general population. It was also better than for essential workers outside the healthcare sector, cumulative dissatisfaction about risky occupation, limited job stability and lack of financial incentives being plausible explanations for this difference [12]. Our intermediate results between these opposite situations could be explained by the Covid outbreak epidemiologic features, our region having experienced a strong but not overwhelming first wave.

The most original feature of this study is the analysis and findings on encouragements and stigmatisation reported by the participants. Indeed, while social media frequently commented on this matter during the first COVID-19 wave, no specific data have been published in the scientific literature to our knowledge. During this survey performed 3 months after the start of the COVID-19 crisis, nearly two-thirds of participants declared having received encouragements, mainly from friends or family, an exceptionally high percentage. Indeed, if healthcare professionals are generally highly regarded by the general public, this had never translated into direct daily messages - such as text messages - with the sole purpose of expressing sympathy as was the case here, with as many as 13% of participants declaring having received personalised gifts (postcards, children drawings, candies and food), something totally unprecedented. In fact, in France, as in many countries, the gratitude of the population towards caregivers was widely expressed during the first COVID-19 wave, in particular because the professional risk was initially poorly contained, leading to severe infections and, in some cases, to death of HCWs, thus recognized by the general population as heroes. Conversely, nearly a fifth of the participants reported stigmatisation over their status as caregivers, an equally unprecedented phenomenon that essentially stemmed from their neighbours’ fear of being infected in a context of very high anxiety in the general population.

The first strength of this study was its large sample size thanks to its proper timing and an easy-to-complete survey. A second asset is that the main categories of the hospital workforce participated in the study in sizeable numbers, making it possible to identify clear differences among occupations. It should be noted however that nurse managers (34% of all nurse managers in our hospital) and physicians (27%) were overrepresented compared to nurses (12%) and nurse assistants (8%) in our study sample. A third strength is the assessment of both professional and personal dimensions of quality of life, in the pre-crisis phase and during the crisis, a feature which seems missing from other published works about quality of life of HCWs during the COVID-19 crisis. Another possible strength is to have largely relied on self-assessments on 0–10 integer scales to assess quality of life, a tool which is familiar to caregivers for the assessment of pain, and which is very easy to use. Conversely, this may be viewed as a limitation when compared to studies that used more codified tools to assess quality of life such as the World Health Organisation tools (WHOQOL-100 and WHOQOL-BREF [13]), the Depression Anxiety Stress Scales (DASS-21, [14]) or the Professional Quality of Life Scale (ProQOL, [15]). Several studies using these scales to assess quality of life among HCWs during the COVID-19 first wave have been published [9, 12, 15–19]. However, while such approaches allowed to quantify stress, anxiety and burnout, they did not offer any qualitative data in contrast to our study. Another limitation stems from the monocentric design of our study, even though our hospital does not have any particular features or a different organisation compared to other university hospitals in France. Finally, it should be noted that findings from this study only apply to the first COVID-19 wave, the subsequent waves having occurred in a markedly different societal context, with the general population possibly less supportive of its caregivers for various reasons, such as the perception of lower professional risk, general weariness, or withdrawal into oneself. In addition, our survey took place at the time the first wave in the Normandy region was strongly decreasing so that HCWs might have been more positive in their evaluation than in previous weeks.

## Conclusions

In our French university hospital, the COVID-19 first wave appeared to have far more impact on a personal than a professional level among caregivers, with nurses and nurse assistants reporting more hardship, feeling less encouraged and more stigmatised. Working in COVID units, even as temporary reinforcement staff, did not have a sizeable negative impact on quality of life, professional satisfaction largely compensating for hardship.

### Final recommendations

Among the three factors that were found independently associated with quality of life change during the COVID-19 first wave, social support in the form of receiving encouragements was the only modifiable factor. This suggests that HCW managers should pay careful attention to the psychological well-being of HCWs and social support should be facilitated. Deserved encouragements are a simple measure with a significant impact on enhancing the well-being of HCWs and are never too many, especially in times of crisis.

## Data Availability

Prof. Jacques Benichou, Head of the Biostatistics and Clinical Research Department at Rouen University Hospital, can be contacted if someone would request additional data available according to French law.

## Abbreviations

HCW: Health Care Worker
QoL: Quality of Life
ICU: Intensive Care Unit
